# Disaster Medicine Training for Medical Students in Lebanon: Quasi-Experimental Comparison of e-Learning and Face-to-Face Modalities

**DOI:** 10.2196/80409

**Published:** 2026-01-28

**Authors:** Ali Msheik, Ruben Peralta, Zeinab Al Mokdad, Christine Bartulec, Linda Abou-Abbas, Dima Anani, Hussein Al Mawla, Hassan Salame, Mohamad Moussa, Awsan Bahattab

**Affiliations:** 1 Faculty of Medical Sciences Lebanese University Beirut, Beyrouth Lebanon; 2 Neurological Surgery Neuroscience Institute Hamad Medical Corporation Doha Qatar; 3 Trauma Surgery Hamad Medical Corporation Doha Qatar; 4 Department of Surgery Universidad Nacional Pedro Henríquez Ureña Santo Domingo Dominican Republic; 5 Medical Ethics Faculty of Medical Sciences Lebanese University Beirut Lebanon; 6 International Committee of the Red Cross Beirut Lebanon; 7 Institut National de Santé Publique, Epidémiologie Clinique et Toxicologie-Liban Beirut Lebanon; 8 Head of the Program Evaluation Committee Faculty of Medical Sciences Lebanese University Beirut Lebanon; 9 Center for Research and Training in Disaster Medicine Università degli Studi del Piemonte Orientale “Amedeo Avogadro” Piemonte Italy

**Keywords:** disaster medicine, education, medical, undergraduate, educational technology, teaching methods, program evaluation, knowledge, surveys and questionnaires, self-efficacy, Lebanon

## Abstract

**Background:**

Despite global advocacy for its integration into medical curricula, disaster medicine (DM) education remains underdeveloped, especially in fragile settings where such training is urgently needed. In Lebanon, a country facing political and economic crises, students face significant barriers to in-person education.

**Objective:**

This study aims to evaluate the effectiveness of e-learning versus face-to-face (F2F) approaches in improving knowledge retention and provides insight into the practical considerations of implementing DM courses in such settings.

**Methods:**

This quasi-experimental study used a Solomon 4-group design to evaluate e-learning and F2F DM courses for second- to fifth-year medical students at the Lebanese University. A total of 205 participants, stratified by academic year, were divided between the 2 modalities. Knowledge was assessed before the course, after the course, and at 1-month follow-up. Confidence, competency, and satisfaction were evaluated after the course using validated tools.

**Results:**

Of 205 participants, 56.6% (n=116) favored e-learning. Both modalities improved knowledge and knowledge retention, with no significant difference between the 2 groups. Fifth-year students achieved the highest gains in knowledge, particularly in the e-learning group. Similarly, no significant difference in satisfaction was observed across modalities, although F2F was preferred overall, except among fifth-year students, who preferred e-learning. Confidence levels were also similar across both modalities, but F2F scored higher for skills like triaging. Feedback emphasized the relevance of the course and advocated for integration of DM into the medical curriculum, and adding practical sessions.

**Conclusions:**

Integrating DM education into the fifth-year curriculum, prior to externship, can enhance preparedness and promote knowledge retention and application in real-world disaster settings. The study shows that e-learning is an effective modality for improving knowledge acquisition and retention in DM. Although feasibility and efficiency were not measured directly, the successful delivery of the course to geographically dispersed students suggests potential practical advantages. Combining F2F practical sessions for specific targeted topics in a blended curriculum is recommended to further enhance medical students’ confidence for future disaster response. These findings support broader policy efforts to institutionalize DM in medical curricula, particularly in fragile and resource-limited settings.

## Introduction

The need for disaster medicine (DM) training in medical education has been recognized since the 1970s, but its integration into the curricula has remained limited until the early 2000s, when DM was incorporated into the curricula of US medical schools. Since then, international frameworks and associations such as the Sendai Framework for Disaster Risk Reduction [1], and the Association of American Medical Colleges and the World Association for Disaster and Emergency Medicine have called for the incorporation of DM and mass casualty incident education in medical schools using scientific evidence-based and flexible approaches [2,3]. Despite these recommendations, limited efforts have been made by universities to implement such training [2].

The lack of DM education has left medical students unprepared to participate in disaster response [4,5]. In contrast, those who have participated in elective DM courses have reported positive results, with high satisfaction, and improved understanding, knowledge, attitudes, and skills toward disaster response [2,6-9]. Still, evaluating these courses remains complicated, due to a lack of standardization across curricula and assessment methods [10,11]. Furthermore, the availability of such training is concentrated in high-income countries and Global North institutes, with limited accessibility for global participation, particularly in fragile and conflict-affected countries, which disproportionately experience higher frequency and impact of disasters [12,13].

Lebanon, home to 5.4 million people and hosting over a million refugees, presents an urgent need for DM education. The country is facing repeated manmade and public health disasters compounded by political instability and an economic crisis, which highlighted the country's fragile health care system [12,14-18]. Yet, undergraduate medical curricula still lack DM education, which remains limited to postgraduate in-service training for practitioners and emergency physicians only. Incorporating DM as a component of the medical curricula constitutes a critical strategy in medical education in Lebanon.

The Lebanese University Faculty of Medical Sciences (LUFMS) is the only public medical school and serves many low-income students all over the country, with around 130 graduates annually. Due to ongoing hostilities and the economic crisis, many low-income students from remote areas are unable to afford the money required to travel to and from campus. The recent COVID-19 pandemic has led to the development of e-learning alternatives that have made it even more accessible to students from these areas. e-learning can provide a cost-effective, equitable, and even practical and interactive solution for DM education [18-24]. However, implementation of such training in crisis-affected settings poses operational challenges [25,26].

This study aimed to evaluate the effectiveness of e-learning versus face-to-face (F2F) approaches in DM education for improving knowledge acquisition and retention among medical students. DM education was delivered through both e-learning and F2F modalities because DM is not included in the undergraduate medical curriculum at LUFMS, and no standardized format for its delivery exists. Evaluating both modalities was therefore essential to determine the most feasible, equitable, and effective approach for nationwide implementation in a context where financial constraints, transportation barriers, and recurrent disruptions frequently hinder in-person attendance. This study also assessed students’ self-perceived confidence, competence, satisfaction, and engagement to inform the acceptability, effectiveness, and feasibility of implementing such courses in fragile or disaster-prone settings.

## Methods

### DM Course Development

The online course used the International Committee of the Red Cross (ICRC) DM e-learning tool. This e-learning tool was an existing, predeveloped ICRC course and was not created by the authors for this study. The development of the ICRC tool was based on the standard principles that are used by multiple courses and learning resources, including the major incident medical management and support course, World Health Organization, and Médecins Sans Frontières, with each module referencing these standards as relevant [27-36]. Development of the F2F course was based on the course contents of the e-learning tool. Both e-learning and F2F DM courses used in this study were based on 10 core topics (Table 1). The training is designed to promote effective learning through interactive, experience-based methods that encourage active engagement and reflection. This approach is informed by constructivist theory, which emphasizes that learners build knowledge through meaningful experiences. To support motivation and a sense of autonomy, the training incorporates principles from self-determination theory, offering flexible learning options that allow individuals to take ownership of their learning process. Additionally, the structure of the content is guided by cognitive load theory, ensuring that information is presented in a clear and manageable way to enhance understanding and retention. The design of the course schedule incorporated a degree of flexibility to accommodate institutional curriculum requirements and the blended e-learning modality used. Of note, principles of responder and scene safety, including self-safety, hazard identification, and ensuring a safe environment before initiating triage or patient care, were incorporated across multiple modules, particularly the introduction to DM, prehospital disaster management, and triage modules, consistent with ICRC, World Health Organization, Médecins Sans Frontières, and major incident medical management and support course standards.

**Table 1:** Topics and subtopics included in the courses in this study.

Topic	Subtopic
Introduction to disaster medicine and taxonomy	Disaster medicine definitions “disaster” and “MCI^a^” Difference between DM^b^ and EM^c^ Public health principles in disasters Disaster management phases: 3 phases The concept of “hazard vulnerability analysis”
Triage in disasters	MCI triage definitions Different triaging systems EM versus DM triaging
Hospital disaster preparation and response	Laws and DM Internal vs external hospital incidents What is an MCI plan? Integration of medical staff
Health consequences of different disasters	Types of disasters and the impact of each Natural vs man-made disasters: the health impact
Prehospital disaster management	MCI: disposition, treatment, and transport Disaster plans and the control chain Functional response rate
Psychosocial care	Psychic reactions and disaster exposure Treatment of incident stress reactions
Presentation of past disasters and public health emergencies	Beirut post explosion COVID-19 pandemic
Pediatric injuries in MCI	Trauma assessment and triage Pediatric-specific injuries Psychological and emotional support Pediatric resuscitation
MCI and burn injuries	Burn severity index Burn resuscitation Wound care and dressing Pain management Psychological support
Management of the dead bodies after disasters	Disaster victim identification Forensic pathology and autopsy Temporary mortuary facilities Body handling Documentation Cultural and religious considerations Public health and hygiene

^a^MCI: mass casualty incident.

^b^DM: disaster medicine.

^c^EM: emergency medicine.

### Study Design

This is a quasi-experimental controlled study using a Solomon 4-group design [37]. Quasi-experimental designs are appropriate when random assignments are impractical, allowing for the evaluation of interventions in real-world settings while maintaining a degree of control over confounding variables [38]. In the Lebanese context, constraints such as transportation difficulties, financial limitations, and geographical barriers rendered random allocation to e-learning and F2F groups unfeasible. Therefore, participants were assigned based on their ability to attend on-campus sessions, considering factors like proximity and access to reliable transportation rather than their personal preference or academic performance, and selection bias related to motivation was minimal. The design balances trade-offs between internal and external validity, feasibility, inclusiveness, and applicability in educational real-life settings in resource-constrained environments [39]. Furthermore, the Solomon 4-group design enhances the robustness of the study by controlling confounding variables and potential pretest sensitization effects [37].

### Target Population

The target population consisted of medical students between the second and fifth academic years. All students from the second to the fifth academic years received identical course content, as DM is not part of the formal curriculum at any level. Standardizing the content ensured comparability across groups and isolated the effect of the delivery modality rather than differences in academic year. No interns were included in this study.

### Sampling

The total number of the targeted population was 512. The sample size was calculated using G*Power software to detect the effect size (Cohen *d*=0.4) for the primary outcome (posttraining effectiveness difference between e-learning and F2F, measured by the significant difference in knowledge scores between the groups), with a significance level of .05, a desired power of 0.80, and a 2-tailed test. The estimated minimum sample size was 174 participants (87 in each group). The population count per group was determined with the assistance of delegates from each year for the medical students via convenience sampling, such as open registration on a first-come, first-served basis, regardless of their academic year. Group assignments were determined based on students’ logistical capabilities. Those with the ability to attend on-campus sessions, considering factors such as proximity to campus, access to reliable transportation, and affordability, were assigned to the F2F cohort. Conversely, students facing logistical barriers that precluded on-campus attendance were placed in the e-learning cohort.

### DM Course Implementation

The study involved delivering a DM course using identical educational materials through 2 modalities: e-learning and F2F sessions. The course was delivered over 3 days per week for 2 weeks. The F2F sessions were implemented by the principal investigator at LUFMS. All synchronous e-learning sessions were also delivered by the principal investigator to ensure standardization across modalities. The digital version for the e-learning groups was a set of modules built onto a learning management system, which was delivered to the respective subgroups in a synchronized fashion by the principal investigator, with the lectures on the same dates to the assigned subgroups. Both groups received a digital version of the respective teaching material at the end of each session. while the F2F group received the slides for the respective session.

### Evaluation Framework and Outcomes

The first 2 levels of Kirkpatrick’s model were used to assess knowledge, knowledge retention, learning, and satisfaction. Additionally, self-efficacy, as a predictor of skill application, was measured through perceived behavioral outcomes, specifically confidence (self-perceived competence), and was guided by social cognitive theory [40].

1. Assessment of the students’ knowledge: Students’ knowledge assessment consisted of 20 multiple-choice questions (Table 2). The 20-item multiple-choice questions assessment included 2 questions per core topic, as summarized in Table 2; the full questionnaire is provided in Multimedia Appendix 1. Each question had 5 possible answers, and only 1 answer was correct.

Based on Solomon 4-group design model, students within each year and learning modality were divided randomly into 2 groups [37]. Each academic year was subdivided into 4 subgroups (A, B, C, and D; Table 3).

**Table 2:** Multiple-choice question topics per core subject.

Topic	Multiple choice questions
Introduction to disaster medicine and taxonomy	Question 1: Which statement is correct about DM^a^?
Triage in disasters	Question 2: What is the purpose of triaging in a medical emergency? Question 3: In the triage system, which color is typically associated with the highest priority level?
Hospital disaster preparedness and response	Question 4: What is the role of MOH^b^ in DM? Question 5: What is an MCI^c^ plan? Question 6: Role of hospitals and prehospital units in preparedness Question 7: Which means of communication is most important in DM?
Health consequences of different disasters	Question 8: Which type of disaster affects the respiratory system? Question 9: Which statement is correct?
Prehospital and hospital disaster management	Question 10: Who controls during a disaster? Question 11: What is the role of an incident commander? Question 12: In an MCI, who is typically responsible for declaring a patient deceased? Question 13: What is the role of hospitals in disaster preparedness?
Psychosocial care	Question 14: What does PTSD^d^ stand for? Question 15: What are treatment modalities for stress during disasters?
Presentation of past disasters and public health emergencies	Question 16: Recall the hospitals damaged during the incident Question 17: What is COVID-19 pandemic most significant impact worldwide?
Pediatric injuries in MCI	Question 18: In an MCI involving pediatric injuries, which of the following is essential to identify first?
MCI and burn injuries	Question 19: In the context of mass casualty incidents involving burn injuries, which aspect is essential for initial assessment?
Management of the dead bodies after disasters	Question 20: When managing dead bodies after disasters, which of the following should be prioritized first?

^a^DM: disaster management.

^b^MOH: Ministry of Health.

^c^MCI: mass casualty incident.

^d^PTSD: posttraumatic stress disorder.

**Table 3:** Subgroup classification of the patients within each academic year and the timing of the assessments based on Solomon 4-group design model.

Time points	Medical students: second-fifth year (subgroups A-D)
First week	Phase I: precourse assessment: Subgroups A and B had face-to-face sessions, and subgroups C and D had e-learning in the first and second weeks
Second week “end of course”	Phase II: postcourse assessment online survey for all subgroups
1 month post course completion	Phase III: assessment for all subgroups

The assessment was done over 3 phases:

- Precourse assessment: conducted 15 minutes before the course for subgroups A and C only.
- Postcourse assessment: conducted after the end of the final session for all subgroups. The pre- and post-assessments used the same questionnaire details.
- Follow-up assessments: conducted 1 month after the finalization of the DM educational course to evaluate knowledge retention.

1. Assessment of the satisfaction and recommendations of students: the assessment of satisfaction was done using a tool developed by Han et al [38]. The tool consisted of questions with a rating scale (Multimedia Appendix 2) [35,37]:
  - “Reaction” of the participants: how they felt and thought about the course
  - “Learning” of the participants: how they described the increase in knowledge after the course.

In addition, 3 open questions addressed the experience of the participants. The first question addressed the most unique aspect of the course. The second question addressed the suggestions of the participants for improvement of the course. The last question requested additional comments from the participants.

1. Evaluation of self-efficacy: a postcourse evaluation of confidence was conducted for all subgroups in each academic year. The evaluation of the confidence level was measured using a validated Disaster Preparedness Evaluation Tool [38,39]. The original version was adapted to the educational material implemented in the course (Multimedia Appendix 3) [35,36]. Students will be asked their level of confidence on a Likert scale as “strongly disagree,” “disagree,” “neutral,” “agree,” and “strongly agree.”

### Data Collection Tools and Methods

The student’s ID, their academic year, and evaluation data were confidentially collected using the Google Forms survey tool, which was converted to a Microsoft Excel sheet and saved anonymously (Multimedia Appendix 4). The handling and access of the data were limited to the principal investigator to ensure data security. All data were stored in accordance with institutional data protection policies.

### Reliability Assessment

A post hoc reliability analysis check of the scale used to assess the knowledge, satisfaction, and confidence level showed excellent internal consistency (Cronbach α was 0.82, 0.92, and 0.963, respectively).

### Data Analysis

#### Quantitative Analysis

Descriptive statistics, including percentages, means, and SD values, were used to describe the sample and summarize survey scores. Knowledge scores were reported as the number of correct responses. Repeated measures ANOVA, also referred to as a within- and between-subjects ANOVA for correlated samples, was used to detect any overall differences between related means at different points in time. Chi-square and Fisher exact tests were used to evaluate differences in the items of the satisfaction scale and confidence scale across the different options. An independent-sample *t* test was used to compare satisfaction and confidence scores in the overall sample and stratified by year of education between the 2 learning modalities. Data were analyzed using the Statistical Package for the Social Sciences (version 27.0; IBM Corp). A *P* value less than .05 was considered significant.

#### Qualitative Analysis

Open question responses were inductively analyzed and coded, and recurrent themes were presented in percentages.

### Ethical Considerations

This project was conducted according to the applied ethical guidelines and received the necessary approvals from the relevant ethical review boards. The study was approved by the Institutional Review Board of Al Zahraa Hospital University Center under reference number 12/2023. Additionally, the study received an exemption from the Ethics Review Board of the ICRC in Geneva. The ICRC Ethics Review Board confirmed that the research proposal presented minimal risks to participants, which were adequately addressed and minimized through the proposed procedures. The exemption reference number for this study is 0623. Informed consent was obtained from all participants, and strict confidentiality was maintained in the handling of all data to ensure participants' privacy and well-being throughout the study.

## Results

### Descriptive Statistics

**Figure 1:**
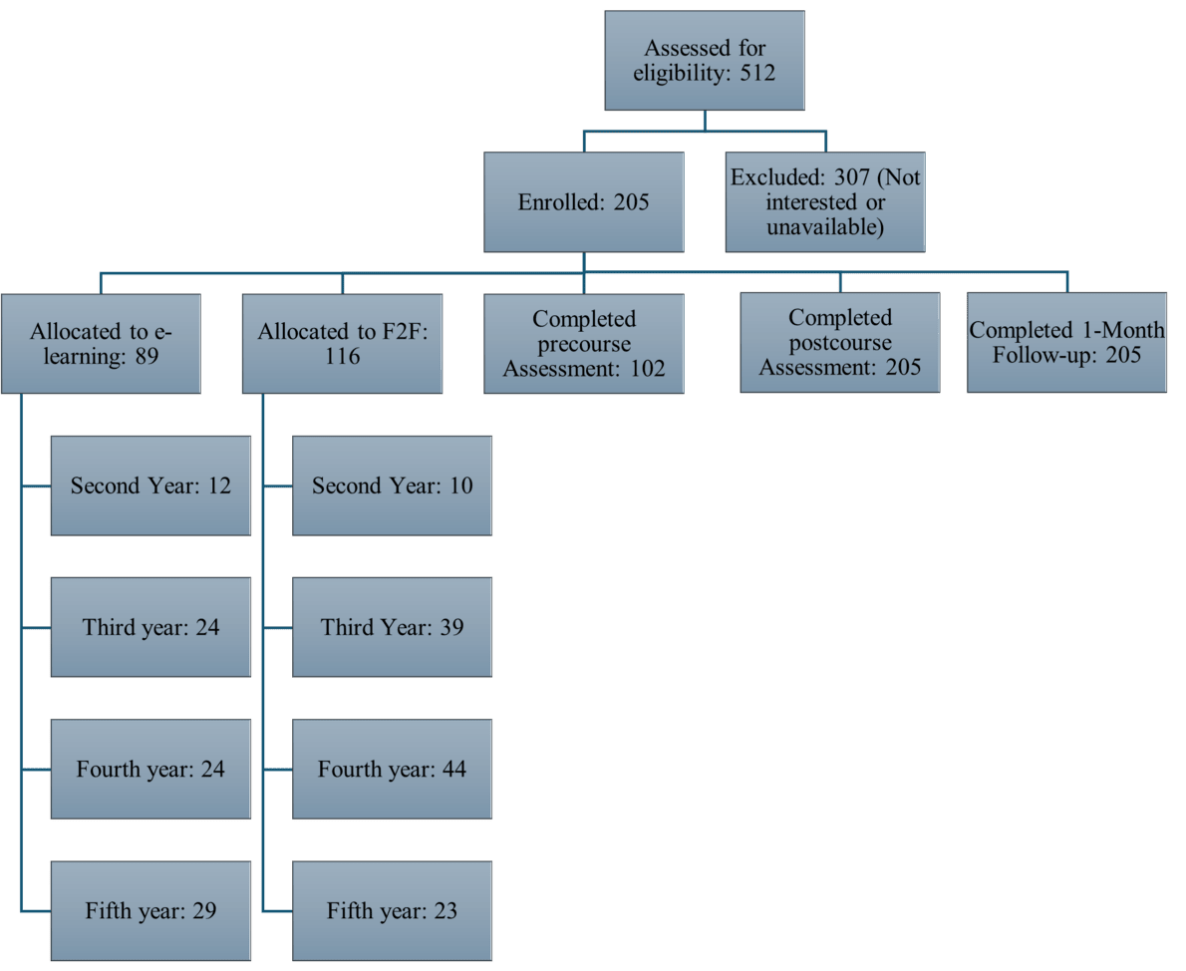
Participant flow diagram showing distribution by academic year and learning modality (F2F vs e-learning). F2F: face-to-face.

Participants were recruited, and the course was conducted between March 2024 and June 2024. The participant flowchart is presented in Figure 1. Out of 512 students at LUFMS from the second to the fifth academic years, 205 (40%) voluntarily participated in the study, of which 116 (56.6%) joined the e-learning cohort, and 89 (43.4%) joined the F2F cohort between March and June 2024. The third and fourth academic year students represented 30.7% (63/205) and 33.2% (68/205) of the participants, respectively. Fifth academic year students were 25.4% (52/205), and the second academic year students were the least (22/205, 10.7%). The distribution of students according to the crosstabulation of the academic year and learning modality is depicted in Figure 1. Participation rates from the second and fifth academic years were comparable across the F2F and e-learning groups, with 12 (13.9%) and 10 (8.6%) students from the second year, and 29 (32.5%) and 23 (19.8%) students from the fifth year, respectively. Nearly 61.9% (39/63) of the third academic year students and 64.7% (44/66) of the fourth academic year students joined the F2F cohort.

### Assessment of Precourse Sensitization

Based on Solomon 4-group design model [37], a summarized comparison for the overall sample is presented in Table 4. The analysis shows that pretested and non-pretested groups demonstrated comparable scores at baseline, at the immediate postcourse assessment, and at 1-month follow-up (*P*=.60, .40, and .64, respectively). These results confirm that the precourse assessment did not introduce sensitization effects or bias the learning outcomes, thereby supporting the internal validity of the study design. Hence, precourse assessment is not a confounding factor and did not affect the outcome across the subgroups.

**Table 4:** Summary of Solomon 4-group analysis for the overall sample.

Overall			Pretest		Posttest		Post 1 month
**F2F^a^**							
	Experimental, mean (SD)	6.3 (2.3)		12.3 (1.8)		14.3 (2.4)	
	Control, mean (SD)	—^b^		12.2 (1.7)		15.7 (3.9)	
**e-Learning**							
	Experimental, mean (SD)	6.6 (2.3)		12 (2.1)		14.6 (3.5)	
	Control, mean (SD)	—		13.5 (3.7)		16.4 (4.4)	
*P* value between groups			.60		.40		.64

^a^F2F: face-to-face.

^b^Not applicable.

### Assessment of the Knowledge Level in the Overall Sample and Across the Academic Years

For the overall sample, both F2F and e-learning groups demonstrated significant improvements from precourse to immediate postcourse and post 1 month, with no significant statistical difference between both modalities overall (*P*=.40 and .64) and across each year (Multimedia Appendix 5). The F2F cohort showed a mean increase of 5.9 (95% CI 5.35-6.45; *P*<.001) and 7.9 (95% CI 7.34-8.46; *P*<.001) from precourse to postcourse and post 1 month, respectively (Table 5). Similarly, the e-learning group exhibited a mean increase of 5.4 (95% CI 4.69-6.11; *P*<.001) and 8 (95% CI 7.11-8.89; *P*<.001) over the same periods. In the second year, the F2F group showed a significant increase of 5.6 (95% CI 3-8.2; *P*=.04) and 7.6 (95% CI 5.68-9.52; *P*=.002) from precourse to postcourse and post 1 month, respectively. However, the e-learning group did not show significant improvement, with a mean increase of 4 (95% CI 0.83-7.17; *P*=.74) and 7 (95% CI 4.57-9.43; *P*=.14), indicating less effectiveness in the second year. Of note, all mean change values were calculated at the individual level and then averaged within each subgroup; no pooled scoring was used.

**Table 5:** Mean change in knowledge scores from precourse to postcourse and 1-month follow-up.

Cohort	Sample size	Postcourse, mean (SD)		1-month follow-up, mean (SD)		*P* value
		Mean (SD)	95% CI	Mean (SD)	95% CI	
F2F total^a^	116	5.9 (3)	5.35-6.45	7.9 (3.1)	7.34-8.46	<.001
e-learning total	89	5.4 (3.4)	4.69-6.11	8 (4.3)	7.11-8.89	<.001
Second-year F2F	10	5.6 (4.2)	3-8.20	7.6 (3.1)	5.68-9.52	.002
Second-year e-learning	12	4 (5.6)	0.83-7.17	7 (4.3)	4.57-9.43	.14
Third-year F2F	39	5.3 (2.1)	4.64-5.96	7.3 (2.3)	6.58-8.02	<.001
Third-year e-learning	24	5.8 (3.2)	4.52-7.08	8.3 (4.3)	6.58-10.02	<.001
Fourth-year F2F	44	7.1 (3.6)	6.04-8.16	9 (3.9)	7.85-10.15	.003
Fourth-year e-learning	24	4.9 (3.3)	3.58-6.22	7.2 (4.8)	5.28-9.12	<.001
Fifth-year F2F	23	6.1 (2.7)	5-7.20	8.2 (3.4)	6.81-9.59	<.001
Fifth-year e-learning	29	6.1 (3)	5.01-7.19	10.1 (2.6)	9.15-11.05	<.001

^a^F2F: face-to-face.

The fifth-year students exhibited the highest mean score increases within their respective learning modalities. The F2F group showed a mean increase of 6.1 (95% CI 5-7.2; *P*=.005) and 8.2 (95% CI 6.81-9.59; *P*<.001) from precourse to postcourse and post 1 month, respectively. The e-learning group showed a comparable increase of 6.1 (95% CI 5.01-7.19; *P*=.005) and 10.1 (95% CI 9.15-11.05; *P*<.001) over the same periods.

### Assessment of the Satisfaction Level in the Overall Sample and Across the Academic Years

The results show that the total satisfaction score (SD) was slightly higher for the F2F cohort (mean 87.6, SD 11.3; 95% CI 85.54-89.66) compared to e-learning (mean 86, SD 14.2; 95% CI 83.05-88.95). Only the e-learning cohort of the fifth academic year students showed a higher score (mean 90.2, SD 11.7; 95% CI 85.94-94.46) compared to F2F (mean 87.5, SD 9.6; 95% CI 83.58-91.42; Table 6). No statistically significant differences in satisfaction scores were observed between F2F and e-learning modalities in the overall sample (*P*=.38) or within any academic year subgroup.

**Table 6:** Mean change in the satisfaction score compared to the precourse assessment: at the postcourse assessment, directly postcourse.

Cohort	Postcourse assessment, mean (SD)	Sample size, n	95% CI	*P* value^a^
F2F^b^ total	87.6 (11.3)	116	85.54-89.66	.38
e-learning total	86 (14.2)	89	83.05-88.95	—^c^
Second-year F2F	87.6 (15)	10	78.3-96.9	.81
Second-year e-learning	86.2 (11.32)	12	79.8-92.6	—
Third-year F2F	87.5 (11.5)	39	83.89-91.11	.51
Third-year e-learning	84.9 (16.65)	24	78.24-91.56	—
Fourth-year F2F	87.6 (11.5)	44	84.2-91	.35
Fourth-year e-learning	84.5 (13.6)	24	79.06-89.94	—
Fifth-year F2F	87.5 (9.6)	23	83.58-91.42	.37
Fifth-year e-learning	90.2 (11.7)	29	85.94-94.46	—

^a^*P* values calculated using independent-sample Welch *t* tests.

^b^F2F: face-to-face.

^c^Not available.

**Figure 2:**
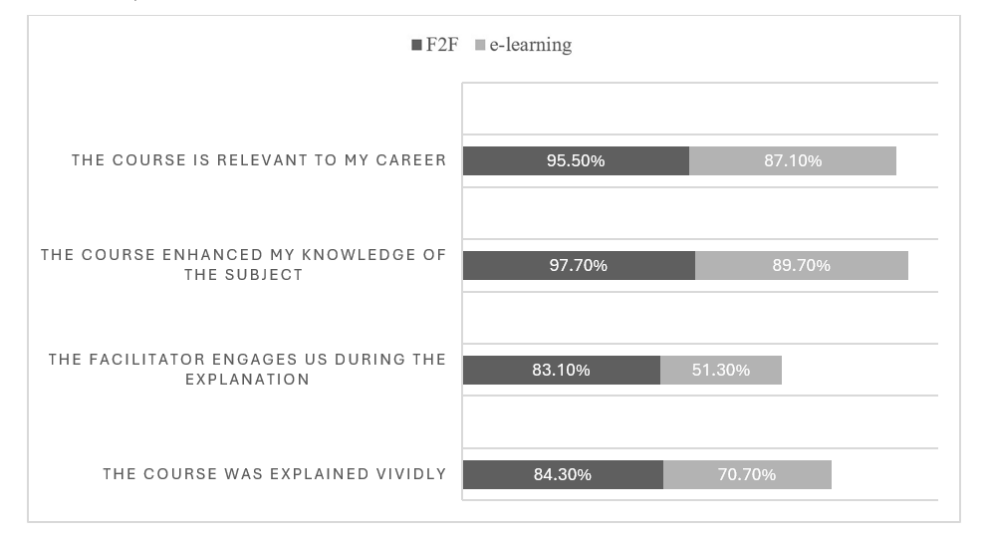
Horizontal bar graph showing the percentage of satisfaction of the participants with each learning modality in the statistically significant questions. F2F: face-to-face.

**Figure 3:**
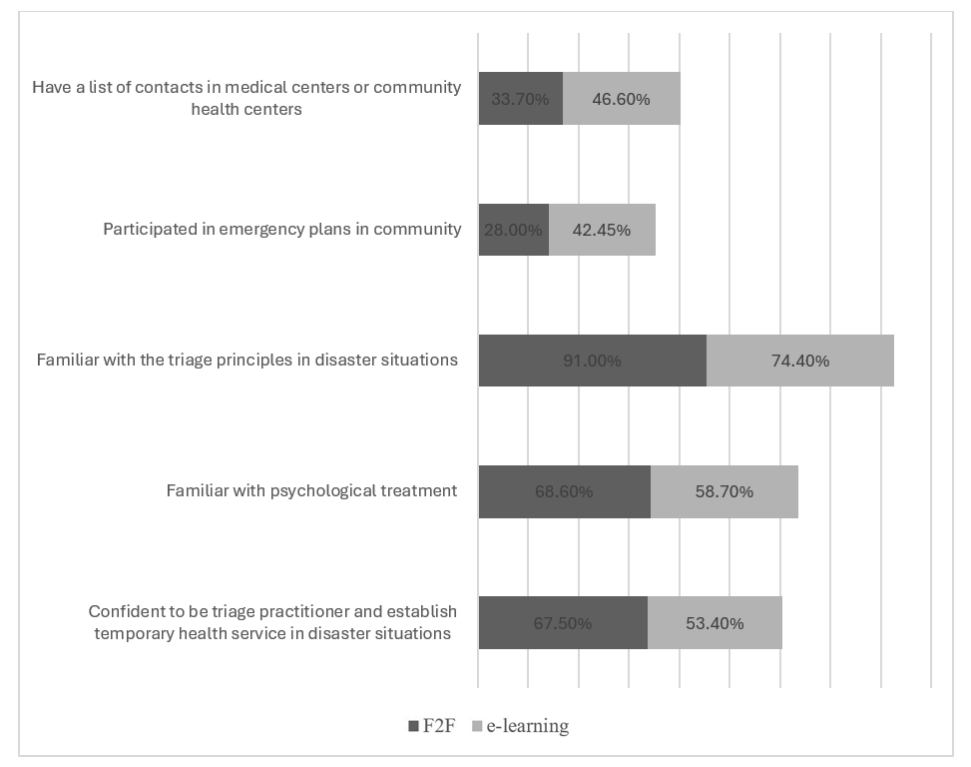
Horizontal bar graph showing the percentage of confidence of the participants with each learning modality in the statistically significant questions. F2F: face-to-face.

Although the comparison of the total satisfaction between F2F and e-learning showed statistically nonsignificant *P* values, comparison of the individual satisfaction scores of each question between the 2 modalities was statistically significant for only 4 questions in favor of F2F (Figure 2). Students were mostly satisfied with the vividness of the course and its relevance to their medical career, enhancement of their knowledge of disaster management, and their engagement during the course (Figure 3).

### Assessment of the Confidence Level in the Overall Sample and Across the Academic Years

The results show that the total confidence score (SD) was similar between F2F (mean 125.5, SD 20.6; 95% CI 121.75-129.25) and e-learning (mean 126.5, SD 23.8; 95% CI 121.56-131.44). Only the F2F cohort of the second academic year students showed scores that were like the overall score, yet better than e-learning, with a mean F2F score of 125.4 (SD 22.5; 95% CI 111.45-139.35) compared to a mean of 116 (SD 13.9; 95% CI 108.14-123.86) for e-learning (Table 7). The e-learning cohort of the fifth academic year students showed higher scores compared to F2F, with a F2F score of mean 124.9 (SD 20.1; 95% CI 116.69-133.11) compared to mean 135.3 (SD 21.71; 95% CI 127.4-143.2) for e-learning. No statistically significant differences in confidence scores were observed between learning modalities in the overall sample (*P*=.75). A marginal trend favoring the e-learning group was noted in the fifth-year subgroup (*P*=.08), although it did not reach statistical significance.

**Table 7:** Mean change in the confidence score compared to the precourse assessment: at the postcourse assessment, directly postcourse.

Cohort	Postcourse assessment, mean (SD)	Sample size, n	95% CI	*P* value^b^
F2F^a^ total	125.5 (20.6)	116	121.75-129.25	.75
e-learning total	126.5 (23.8)	89	121.56-131.44	—^c^
Second-year F2F	125.4 (22.5)	10	111.45-139.35	.27
Second-year e-learning	116 (13.9)	12	108.14-123.86	—
Third-year F2F	124.7 (23.1)	39	117.45-131.95	.99
Third-year e-learning	124.6 (28.4)	24	113.24-135.96	—
Fourth-year F2F	127 (18.7)	44	121.47-132.53	.70
Fourth-year e-learning	125 (21.1)	24	116.56-133.44	—
Fifth-year F2F	124.9 (20.1)	23	116.69-133.11	.08
Fifth-year e-learning	135.3 (21.71)	29	127.4-143.2	—

^a^F2F: face-to-face.

^b^*P* values calculated using independent-sample Welch *t* tests.

^c^Not available.

Although the comparison of the total confidence between F2F and e-learning showed no statistically significant *P* values (Table 7, the comparison of the individual confidence score of each question between the 2 modalities was statistically significant for only 5 questions. Students were mostly confident about their knowledge of triaging principles in a disaster through F2F learning (105/116, 91%) versus e-learning (66/89, 74.4%). Similarly, students expressed a higher familiarity with psychological treatment and the ability to triage patients in disaster situations through F2F learning (80/116, 68.6% and 78/116, 67.5%, respectively) relative to e-learning (52/89, 58.7% and 48/89, 53.4%, respectively). The confidence level was below 50% for either modality when students were asked about participation in community emergency plans and having contacts in community health centers.

### Unique Aspect of the Course

The participants highlighted the distinctiveness of the course. Around 85% (174/205) of the participants appreciated the relevance of the course to real-life situations and disaster management. Nearly 75% (153/205) of the participants found engagement with a variety of topics, including mass casualty incidents, triaging, and the management of specific disasters like white phosphorus. The innovative teaching methods and the adaptation of the course to the Lebanese situation were praised by 70% (143/205) of the participants, while 65% (133/205) valued the case studies that helped to effectively manage the disasters.

### Areas of Improvement

Around 60% (123/205) of the participants suggested the inclusion of interactive and practical sessions to reinforce through hands-on practice. Nearly 55% (112/205) of the participants suggested improvement of the scheduling of the sessions, while some proposed that it should become a mandatory part of the curriculum. Half of the participants noted that the artificial intelligence voice used in the e-learning course was mildly distracting. Around 45% (92/205) suggested that live sessions would enhance the course’s effectiveness, especially for complex medical terms and scenarios.

### Additional Comments

About 80% (164/205) of the participants expressed appreciation for the course and gratitude for the opportunity to learn about disaster management. Nearly 70% (143/205) recognized the importance of the course, considering the recent events in Lebanon. A desire for the incorporation of similar courses into the curriculum was noted by 65% (133/205) of the participants. There were also suggestions from 55% (112/205) of participants to expand the course to include more practical exercises, community-based projects, and timely distribution of course materials to ensure better knowledge retention.

## Discussion

### Principal Findings

This study evaluated whether e-learning is comparable to F2F instruction in DM training for LUFMS medical students and contributes to a growing body of evidence supporting the effectiveness of e-learning [40-45]. The results demonstrated significant improvements in knowledge and retention for both modalities, with no statistical difference between them (*P*=.40). However, F2F training resulted in higher confidence in practical skills like triage (91% vs 74.4%), while e-learning resulted in superior retention among fifth-year students. Satisfaction was high in both groups, although qualitative feedback emphasized the need for more hands-on sessions (60%). The results have direct implications for the delivery of DM education and the expectation of outcomes, especially in countries of protracted conflict like Lebanon [46,47]. A blended DM training approach with the skill-building benefits of targeted F2F sessions offers the most pragmatic solution. Still, the equivalence in knowledge gains supports e-learning as a viable alternative in resource-limited settings.

While both delivery methods were effective across academic years, the higher mean gains in knowledge and retention observed among fifth-year e-learning students can be attributed to their advanced medical knowledge compared to students in the preceding academic years. The LUFMS curriculum typically incorporates diagnostic courses within the fifth year, and students commonly undertake a 6-week externship after the fifth academic year. This confluence of advanced knowledge and practical experience may have motivated the fifth-year students to excel in the assessments, recognizing the opportunity to apply their learning in real-life and clinical settings [6]. Additionally, the fifth academic year students were the only group who reported satisfaction and confidence levels with e-learning higher compared to F2F due to their 2-year antecedent exposure to online learning. Given their advanced medical knowledge and diagnostic expertise, the students’ ability to integrate prior knowledge with new concepts acquired through e-learning and independent research likely played a significant role in their favorable outcomes.

Both modalities showed low confidence in community emergency plan participation and health center contacts. This can be attributed to the fact that the participants have not engaged in hospital situations and do not interact with hospital staff except their academic mentors. Therefore, they have limited knowledge about the emergency plans in hospitals. This suggests DM implementation should be timed with clinical rotations to maximize its relevance and effectiveness.

### Limitations

This study has several limitations, which stem from the difficulty of conducting similar studies in a volatile context such as Lebanon, a country with a history of disasters and emergencies, which also impacts the educational settings. Nevertheless, the findings are particularly relevant and have direct application and impact in practice. Voluntary recruitment and the lack of random assignments of the participants may have compromised internal validity, although the real-world applicability, along with sufficient sample size, enhances the study’s external validity, especially for comparable settings. Furthermore, the application of the Solomon 4-group design model classification of the participants allowed for control confounding and pretest sensitization [37], enhancing the internal validity of the study. The short follow-up period limits the conclusion about long-term retention. Still, the significant increase in knowledge over time suggests the consolidation of learning over time due to its real application in their setting. Finally, the reliance on self-reported learning and confidence can introduce bias, but the results were also triangulated by qualitative data and objective assessment with a validated tool [48-51], which also enhances the applicability and internal validity of the study.

Although “lack of interest” was the most frequently selected reason for nonparticipation, this explanation likely underrepresents the true contextual barriers faced by students. Informal feedback indicated that transportation costs, fuel shortages, intermittent electricity and internet access, work obligations, and exam schedules limited their ability to participate. Therefore, the high nonparticipation rate should be interpreted within Lebanon’s unstable socioeconomic context, where such logistical constraints, not genuine disinterest in DM, are more plausible explanations for nonenrollment.

### Conclusions

This study demonstrates that e-learning and F2F DM education are comparably effective in improving knowledge retention, confidence, and perceived competence among Lebanese medical students. The findings support integrating a DM course into the fifth-year curriculum before externships to enhance preparedness and application of skills. A blended approach combining theoretical e-learning with practical F2F sessions may further strengthen practical competencies. These results provide evidence to guide curriculum development and DM training implementation at the LUFMS.

## Appendix

Multimedia Appendix 1 Questions for knowledge for phases I, II, and III.

Multimedia Appendix 2 Questions for the evaluation of the satisfaction of participants in the DM course utilizing the Likert scale.

Multimedia Appendix 3 Questions for evaluations of the confidence of participants in the disaster management educational course utilizing the Likert scale.

Multimedia Appendix 4 All data collection schemes.

Multimedia Appendix 5 Mean knowledge scores for the four groups over the three assessment phases in the overall sample and stratified by year of education and according to the Solomon 4-group design.

## Abbreviations

DM: disaster medicine
LUFMS: Lebanese University Faculty of Medical Sciences
F2F: face-to-face
ICRC: International Committee of the Red Cross

## Data Availability

Data are available with the corresponding author upon request.
