# Supplementary material for: Disaster Medicine Training for Medical Students in Lebanon: Quasi-Experimental Comparison of e-Learning and Face-to-Face Modalities
Source: JMIR Med Educ. 2026 Jan 28;12:e80409. doi: 10.2196/80409 (PMC12895154; doi:10.2196/80409)
Supplement: Multimedia Appendix 1 [file mededu_v12i1e80409_app1.pdf]

Implementation of Disaster Medicine Course for Medical Students of the Faculty of Medical Sciences of the Lebanese University: A Pilot Randomized Educationa Controlled Study

Phase I: Pre-course Assessment
This is a multiple choice question-survey (MCQ) aimed to:

1. Assess the pre-course knowledge of the medical students of the Lebanese University about Disaster Management
2. Evaluate the perception of disaster management by the medical students of the Lebanese University

* Indicates required question

1. Principal Investigator: Dr. Ali Msheik

Purpose of the Study:

The purpose of this study is to develop and implement a disaster management course for medical Lebanese University students. In parallel with the implementation, there is a need t evaluate the effectiveness of this implementation that can be evaluated by measuring students' mastery of knowledge and assessing their satisfaction with the course.

Study Procedures:

Participants will be randomly assigned to either a study group or a control group. The study group will receive additional disaster medicine education as part of their regular curriculum. Participants in both groups will be asked to complete surveys to assess their knowledge an skills related to disaster preparedness.

Risks and Benefits:

There are no known risks associated with participating in this study. Participants may benef from increased knowledge and skills related to disaster preparedness, which may enhance their ability to respond to emergencies in their future careers.

Confidentiality:

All data collected as part of this study will be kept confidential and will only be accessed by

authorized members of the research team.

Voluntary Participation:

Participation in this study is voluntary, and participants may withdraw at any time without penalty or loss of benefits to which they are otherwise entitled.

Contact Information:

If you have any questions about this study, please contact Ali Msheik, at dr.alimsheik@gmail.com /009613927867.

Signature:

By selecting **yes**, I acknowledge that I have read and understand the information provided i this informed consent form and agree to participate in this study.
Mark only one oval.

- Yes
- No

2. Which group are you ? *

Mark only one oval.

- F2f
- E-learning

3. Did you do the pre-assessment exam before the course? *

Mark only one oval.

- yes
- no

Academic Year Choice

Your choice is mandatory to allow segregation of participants for inter-academic year comparison.

4. **Which academic year are you in?** *

Mark only one oval.

- Second
- Third
- Fourth
- Fifth

Assessment of the pre-course knowledge of the medical students of the Lebanese University about Disaster Management

This section comprises 20 questions. It is allocated 30 minutes.

5. **Which definition best describes the term "disaster"?** * 0 pc

Mark only one oval.

- a sudden accident or a natural catastrophe that causes great damage or loss of life
- the condition where casualties overwhelm a healthcare system's capacity to manage using it[illegible] resources
- a flood, hurricane or earthquake
- an accident with more than 100 casualties
- an incident that occurs when a chemical or other substance that may harm people's health has been released into the air, water, or ground

6. **In disaster medicine , what does an MCI stand for?** * 0 pc

Mark only one oval.

- A mass casualty incident (MCI)
- Mild cognitive impairment (MCI)
- Media Control Interface (MCI)
- An incident with more than 60 casualities.
- Mortality Count Index (MCI)

7. **Which of the following is NOT a public health principle in disasters?** * 0 pc

Mark only one oval.

- Prepardness
- Risk assessment
- Communciation
- Recovery and Rehabilitation
- Budget allocation

8. **Which of the following represents the correct sequence of the disaster management phases?** * 0 pc

Mark only one oval.

- Preparation, Response, Mitigation, Recovery, Prevention
- Prevention, Mitigation, Preparation, Response, Recovery
- Response, Recovery, Mitigation, Prevention, Preparation
- Mitigation, Prevention, Response, Preparation, Recovery
- Prevention, Response, Mitigation, Recovery, Preparation

9. **What is the difference between a hazard and a risk?** * 0 pc

Mark only one oval.

- Hazards are potential sources of harm, while risks are the likelihood of harm occurring.
- Risks are potential sources of harm, while hazards are the likelihood of harm occurring.
- Hazards and risks are the same thing and can be used interchangeably.
- Hazards are things that can be controlled, while risks cannot.
- Risks are things that can be controlled, while hazards cannot.

10. **What is triaging in the context of emergency medical response?** * 0 pc

Mark only one oval.

- The process of identifying and prioritizing patients based on the severity of their injuries or illnesses.
- The process of transporting patients to the hospital as quickly as possible.
- The process of administering life-saving treatments to patients in critical condition.
- The process of providing emotional support to patients and their families.
- The process of determining the cause of a medical emergency.

11. **What is the main difference between triaging in disaster management (DM) and emergency management (EM)?** * 0 pc

Mark only one oval.

- There is no difference; triaging is the same in both DM and EM.
- Triaging in DM is focused on identifying and prioritizing resources, while triaging in EM is focused on identifying and prioritizing patients.
- Triaging in DM is focused on identifying and prioritizing patients, while triaging in EM is focused on identifying and prioritizing resources.
- Triaging in DM is only used in natural disasters, while triaging in EM is used in all types of emergencies.
- Triaging in DM is only used in man-made disasters, while triaging in EM is used in natural disasters.

12. **What is secondary triaging in emergency medical response?** * 0 pc

Mark only one oval.

- The process of prioritizing patients based on the severity of their injuries or illnesses.
- The process of identifying patients who require immediate life-saving interventions.
- The process of providing ongoing care and treatment to patients during transport to the hospital.
- The process of re-evaluating and adjusting patient priorities as their conditions change.
- The process of transporting patients to the hospital after initial triage and stabilization.

13. **In emergency medical response, which color is typically matched with each triage code?** * 0 pc

Mark only one oval.

- Green - Immediate
- Yellow - Delayed
- Red - Minimal
- Black - Expectant
- White - Minimal

14. **In a mass casualty incident (MCI), who is typically responsible for declaring a patient deceased?** * 1 p

Mark only one oval.

- Emergency medical responders on the scene
- The hospital staff upon arrival of the patient at the hospital.
- The patient's family or next of kin.
- The police officers present at the scene of the incident.
- A coroner or medical examiner.

15. **What is the role of the Ministry of Health (MOH) in disaster management?** *

Mark only one oval.

- Providing immediate search and rescue operations during emergencies.
- Coordinating with international organizations to provide disaster relief.
- Developing and implementing emergency preparedness plans and protocols.
- Leading post-disaster reconstruction and rehabilitation efforts.
- Providing emergency financial assistance to affected individuals and families.

16. **What is an MCI plan in disaster management?** *

Mark only one oval.

- A plan that outlines the roles and responsibilities of first responders during an emergency.
- A plan that provides guidelines for providing emergency medical care to victims of a mass casualty incident.
- A plan that details the steps for assessing and managing the physical and emotional needs of survivors after a disaster.
- A plan that outlines procedures for evacuating individuals and providing shelter during a disaster.
- A plan that establishes communication channels and protocols for coordinating response efforts during a disaster.

17. **What is the role of hospitals in disaster preparedness?** *

Mark only one oval.

- Providing immediate search and rescue operations during emergencies.
- Coordinating with international organizations to provide disaster relief.
- Developing and implementing emergency preparedness plans and protocols.
- Leading post-disaster reconstruction and rehabilitation efforts.
- Providing emergency financial assistance to affected individuals and families.

18. **Which means of communication is considered the most important in disaster management?**

Mark only one oval.

- Landline phones
- Cellphones
- Radio communication
- Internet-based communication
- Social media platforms

19. **Which type of disaster is most likely to affect the respiratory system?** *

Mark only one oval.

- Flood
- Heatwave
- Earthquake
- Chemical spill
- Power outage

20. **During a disaster, who is responsible for overall coordination and control?** *

Mark only one oval.

- The federal government
- The state government
- The local government
- The Red Cross
- The United Nations

21. **What is the primary role of an incident commander during a disaster?** *

Mark only one oval.

- To provide medical care to victims
- To coordinate communication between agencies
- To manage and direct the response effort
- To provide counseling to survivors
- To conduct damage assessments

22. **What does PTSD stand for?** *

Mark only one oval.

- Post-traumatic stress diagnosis
- Post-traumatic stress disorder
- Post-traumatic stress development
- Post-traumatic stress debriefing
- Post-traumatic self-diagnosis

23. **What are some treatment modalities that may be used to address stress and trauma during and after a disaster?**

Mark only one oval.

- Cognitive behavioral therapy
- Mindfulness meditation
- Eye movement desensitization and reprocessing (EMDR)
- All of the above
- None of the above

24. **What has been the most significant impact of the COVID-19 pandemic worldwide?** *

Mark only one oval.

- Economic recession
- Mental health crisis
- Strained healthcare systems
- Travel restrictions and border closures
- All of the above

This content is neither created nor endorsed by Google.

Google Forms

Implementation of Disaster Medicine Course for Medical Students of the Faculty of Medical Sciences of the Lebanese University: A Pilot Randomized Educationa Controlled Study

Phase II: Post-course Assessment
This is a multiple choice question-survey (MCQ) aimed to:

1. Assess the post-course knowledge of the medical students of the Lebanese University about Disaster Management
2. Evaluate the perception of disaster management by the medical students of the Lebanese University and their impression about implementation of a disaster management course in the teaching curriculum

* Indicates required question

1. Principal Investigator: Dr. Ali Msheik

Purpose of the Study:

The purpose of this study is to develop and implement a disaster management course for medical Lebanese University students. In parallel with the implementation, there is a need t evaluate the effectiveness of this implementation that can be evaluated by measuring students' mastery of knowledge and assessing their satisfaction with the course.

Study Procedures:

Participants will be randomly assigned to either a study group or a control group. The study group will receive additional disaster medicine education as part of their regular curriculum. Participants in both groups will be asked to complete surveys to assess their knowledge an skills related to disaster preparedness.

Risks and Benefits:

There are no known risks associated with participating in this study. Participants may benef from increased knowledge and skills related to disaster preparedness, which may enhance their ability to respond to emergencies in their future careers.

Confidentiality:

All data collected as part of this study will be kept confidential and will only be accessed by

authorized members of the research team.

Voluntary Participation:

Participation in this study is voluntary, and participants may withdraw at any time without penalty or loss of benefits to which they are otherwise entitled.

Contact Information:

If you have any questions about this study, please contact Ali Msheik, at dr.alimsheik@gmail.com /009613927867.

Signature:

By selecting **yes**, I acknowledge that I have read and understand the information provided i this informed consent form and agree to participate in this study.

Mark only one oval.

- Yes
- No

2. Which group are you ? *

Mark only one oval.

- F2f
- E-learning

3. Did you do the pre-assessment exam before the course? *

Mark only one oval.

- yes
- no

Academic Year Choice

Your choice is mandatory to allow segregation of participants for inter-academic year comparison.

4. **Which academic year are you in?** *

Mark only one oval.

- Second
- Third
- Fourth
- Fifth

Assessment of the post-course knowledge of the medical students of the Lebanese University about Disaster Management

This section comprises 20 questions. It is allocated 30 minutes.

5. **Which of the statements is true?** *

Mark only one oval.

- A disaster is a catastrophic event that results in widespread damage and loss of life which is beyond the capacity of the healthcare system to contain
- MCI stands for major critical injury
- A principle of public health in disasters is focusing resources on individuals who are able to pay for medical care
- The correct sequence of the disaster management phases is "Prevention, Response, Mitigation, Recovery, Preparation"
- A hazard is a natural disaster, while a risk is a man-made disaster.

6. **What is the purpose of triaging in a medical emergency?** *

Mark only one oval.

- To determine the cause of the emergency
- To administer first aid to the patient
- To transport the patient to the hospital
- To sort patients based on the severity of their condition
- To provide emotional support to the patient

7. **In the triage system, which color is typically associated with the most severe priority level**

Mark only one oval.

- Green
- Yellow
- Red
- Black
- Blue

8. **In a mass casualty incident (MCI), who is typically responsible for declaring a patient deceased?**

Mark only one oval.

- The first responder on the scene
- The triage officer
- The attending physician at the hospital
- The medical examiner
- The patient's family members

9. **What is the role of the Ministry of Health (MOH) in disaster management?** *

Mark only one oval.

- To provide immediate emergency medical care to victims of a disaster
- To coordinate the response efforts of healthcare providers and facilities in the affected area
- To conduct research on disaster medicine and develop new treatment protocols
- To assess the effectiveness of disaster preparedness plans and make recommendations for improvement
- To provide financial assistance to victims of a disaster and their families

10. **What is an MCI plan in disaster management?** *

Mark only one oval.

- A plan for managing medical emergencies in a hospital or healthcare facility
- A plan for responding to natural disasters such as hurricanes and earthquakes
- A plan for providing mental health services to victims of a disaster
- A plan for coordinating the efforts of multiple agencies and organizations in response to a mass casualty incident
- A plan for preventing the spread of infectious diseases in a community

11. **What is the role of hospitals in disaster preparedness?** *

Mark only one oval.

- To provide immediate emergency medical care to victims of a disaster
- To conduct research on disaster medicine and develop new treatment protocols
- To coordinate the response efforts of healthcare providers and facilities in the affected area
- To assess the effectiveness of disaster preparedness plans and make recommendations fo improvement
- To establish and maintain emergency management plans and protocols to ensure continuit of patient care during and after a disaster

12. **Which means of communication is considered the most important in disaster management?**

Mark only one oval.

- Email communication
- Telephone communication
- Radio communication
- Social media communication
- Face-to-face communication

13. **Which statement is correct?** *

Mark only one oval.

- Volcanic eruptions are man-made disasters
- Chemical gases affect the digestive system
- Explosions are natural disasters
- The respiratory system can be affected by radiation disasters

14. **What is the primary role of an incident commander during a disaster?** *

Mark only one oval.

- To provide medical care to victims
- To coordinate communication between agencies
- To make policy decisions regarding disaster response
- To lead search and rescue efforts
- To oversee overall response and control of the incident

15. **Which of the following options correctly defines PTSD?** *

Mark only one oval.

- A mental disorder caused by the experience of a traumatic event
- A physical disorder caused by exposure to chemicals or radiation
- A genetic disorder that affects the body's ability to fight infections
- A disorder caused by the excessive use of drugs or alcohol
- A neurological disorder caused by head injuries or stroke

16. In a Mass Casualty Incident (MCI) involving pediatric injuries, which of the following is essential to identify first?

Mark only one oval.

- Vital sign values for pediatrics
- Triaging of cases
- Type of triage
- Surgery
- Acupuncture

17. In the context of Mass Casualty Incidents (MCI) involving burn injuries, which aspect is essential for initial assessment?

Mark only one oval.

- Role of oral hydration on the field
- Calculation of the percentage of burnt area
- Pain control versus resuscitation
- Requirements of the burn patient
- None of the above

18. When managing dead bodies after disasters, which of the following should be prioritized first?

Mark only one oval.

- Identification and documentation of the deceased
- Implementing measures of hygiene
- Transportation of bodies to a temporary morgue
- Communication with family members

Evaluation of the perception of disaster management by the medical students of the Lebanese University and their impression about implementation of a disaster management course in the teaching curriculum

This section comprises of 23 statements. It is allocated 20 minutes.
The choices are based on a Likert scale design.

19. I participate in one of the following educational activities on regular basis continuing education classes, seminars, or conferences dealing with disaster preparedness.

Mark only one oval per row.

	Strongly Agree	Agree	Neutral	Disagree	Strongly Disagree
**I am able to discern the signs and symptoms of acute stress disorder and post traumatic stress syndrome (PTSD)**					
**The course met my expectations**					
**The course was organized**					
**The course duration was right**					
**The course urged me to search the elaborated topics**					
**The facilitator is friendly**					
**The course was explained vividly**					
**The facilitator engages us during the explanation**					
**The course enhanced my knowledge of the subject**					
**I would recommend this course to my colleagues**					
**The course is relevant to my carrier**					
**Do you feel more confident about dealing with disasters after this course?**					

**I would feel confident providing education on coping skills and training for patients who experience traumatic situations so they are able to manage themselves**	◯	◯	◯	◯	◯
**I have participated in emergency plan drafting and emergency planning for disaster situations in my community.**	◯	◯	◯	◯	◯
**I participate in disaster drills or exercises at my workplace (clinic, hospital, etc.) on a regular basis.**	◯	◯	◯	◯	◯
**I have a list of contacts in the medical or health community in which I practice. I know referral contacts in case of a disaster situation (health department, e.g.,).**	◯	◯	◯	◯	◯
**Finding relevant information about disaster preparedness related to my community needs is an obstacle to my level of preparedness**	◯	◯	◯	◯	◯
**I know where to find relevant research or information related to disaster preparedness and management to fill in gaps in my knowledge.**	◯	◯	◯	◯	◯
**I consider myself prepared for the management of disasters.**	◯	◯	◯	◯	◯
**I find that the research literature on disaster preparedness and management is easily accessible.**	◯	◯	◯	◯	◯
**I find that the research literature on disaster preparedness is understandable.**	◯	◯	◯	◯	◯
**I am familiar with the local emergency response system for disasters.**	◯	◯	◯	◯	◯
**I know who to contact (chain of command) in disaster situations in my community.**	◯	◯	◯	◯	◯

20. Answer on a Likert scale basis to evaluate yourself after the course versus your knowledg before the course

Mark only one oval per row.

	Strongly agree	agree	neutral	disagree	strongly disagree
**Confident to take care of patients without supervision**					
**Confident to educate patients on stress related to injury**					
**Confident to be triage nurse practitioner and establish temporary health service in disaster situations**					
**Confident as a manager or coordinator in shelter.**					
**Able to recognize the signs and symptoms of acute stress disorder and post-traumatic stress disorder (PTSD)**					
**Confident to provide education on coping skills**					
**Confident to perform as a first responder**					
**Confident to perform health assessment in case of bioterrorism**					
**Familiar to perform role of nurse practitioner in a post-disaster situatio**					
**Confident to implement emergency plan and evacuation**					
**Managing emotional outcomes for acute stress disorder or PTSD**					
**Familiar with the roles of organizations in disaster response**					
**Familiar to perform health assessment for PTSD**					

Participate in peer evaluation of skills on disaster preparedness and response.					
Able to describe role in response phase					
Familiar with psychological treatment					
Confident to perform isolation procedure to reduce risk of community exposure					
Know how to operate the decontamination procedures					
Know how to use personal protective equipment					
Familiar with the triage principles in disaster situations					
Have personal/family emergency plans					
Familiar with the local emergency response system					
Have an agreement with family members					
Able to manage the common reactions of disaster survivors					
Participated in emergency plans in community					
Participated in educational activities about disaster preparedness					
Participated in creating an emergency plan for improvements on the local or national level					
Read journals related to disaster preparedness					
Be considered a key leader in the community in a disaster situation					

Know the chain of command in the community					
Have a list of contacts in medical centers or community health centers					
Know the limitations in knowledge and skills about disaster situations					
Know sources of information related to disaster preparedness					
Interested in classes about disaster preparedness in the community					
The research literature on disaster preparedness is easily accessible					
Understand research literature on disaster preparedness					

21. **What was the most unique about this course?** *

22. **What can be improved in the future?** *

23. **Any additional comments?** *

This content is neither created nor endorsed by Google.

Google Forms

Implementation of Disaster Medicine Course for Medical Students of the Faculty of Medical Sciences of the Lebanese University: A Pilot Randomized Educationa Controlled Study

Phase III: Post-course Assessment
This is a multiple choice question-survey (MCQ) aimed to:

1. Assess the post-course knowledge of the medical students of the Lebanese University about Disaster Management
2. Evaluate the perception of disaster management by the medical students of the Lebanese University and their impression about implementation of a disaster management course in the teaching curriculum

* Indicates required question

1. Principal Investigator: Dr. Ali Msheik

Purpose of the Study:

The purpose of this study is to develop and implement a disaster management course for medical Lebanese University students. In parallel with the implementation, there is a need t evaluate the effectiveness of this implementation that can be evaluated by measuring students' mastery of knowledge and assessing their satisfaction with the course.

Study Procedures:

Participants will be randomly assigned to either a study group or a control group. The study group will receive additional disaster medicine education as part of their regular curriculum. Participants in both groups will be asked to complete surveys to assess their knowledge an skills related to disaster preparedness.

Risks and Benefits:

There are no known risks associated with participating in this study. Participants may benef from increased knowledge and skills related to disaster preparedness, which may enhance their ability to respond to emergencies in their future careers.

Confidentiality:

All data collected as part of this study will be kept confidential and will only be accessed by

authorized members of the research team.

Voluntary Participation:

Participation in this study is voluntary, and participants may withdraw at any time without penalty or loss of benefits to which they are otherwise entitled.

Contact Information:

If you have any questions about this study, please contact Ali Msheik, at dr.alimsheik@gmail.com /009613927867.

Signature:

By selecting **yes**, I acknowledge that I have read and understand the information provided i this informed consent form and agree to participate in this study.

Mark only one oval.

- Yes
- No

2. Which group are you ? *

Mark only one oval.

- F2f
- E-learning

3. Did you do the pre-assessment exam before the course? *

Mark only one oval.

- yes
- no

Academic Year Choice

Your choice is mandatory to allow segregation of participants for inter-academic year comparison.

4. **Which academic year are you in?** *

Mark only one oval.

- Second
- Third
- Fourth
- Fifth

Assessment of the post-course knowledge of the medical students of the Lebanese University about Disaster Management

This section comprises 20 questions. It is allocated 30 minutes.

5. **Which definition best describes the term "disaster"?** *

Mark only one oval.

- An unexpected event that causes minor disruption to daily life
- A natural occurrence that does not have a significant impact on society
- A catastrophic event that results in widespread damage and loss of life which is beyond the capacity of the healthcare system to contain
- A situation that leads to the loss of property, but not human lif
- A temporary setback that can be easily managed and resolved

6. **Which of the following best describes what MCI stands for in disaster medicine?** *

Mark only one oval.

- Mass Casualty Incident
- Multiple Catastrophic Injuries
- Medical Critical Incident
- Major Critical Injury
- None of the above

7. **Which of the following is a principle of public health in disasters?** *

Mark only one oval.

- Providing psychological support for disaster victims
- Treating patients with the most severe injuries first
- Budget allocation
- Focusing resources on individuals who are able to pay for medical care
- Prioritizing care for individuals based on their age and social status

8. **Which of the following represents the correct sequence of the disaster management phases?**

Mark only one oval.

- Response, Recovery, Mitigation, Prevention, Preparation
- Preparation, Response, Mitigation, Recovery, Prevention
- Prevention, Response, Mitigation, Recovery, Preparation
- Prevention, Mitigation, Preparation, Response, Recovery
- Mitigation, Prevention, Response, Preparation, Recovery

9. **What is the difference between a hazard and a risk?** *

Mark only one oval.

- A hazard is the likelihood of harm occurring, while a risk is a potential source of harm.
- A hazard is a type of disaster, while a risk is a type of emergency.
- A hazard is a natural disaster, while a risk is a man-made disaster.
- A hazard is a specific event, while a risk is a general threat to safety.
- A hazard is a potential source of harm, while a risk is the likelihood of that harm occurring.

10. **What is the purpose of triaging in a medical emergency?** *

Mark only one oval.

- To administer first aid to the patient
- To transport the patient to the hospital
- To sort patients based on the severity of their condition
- To determine the cause of the emergency
- To provide emotional support to the patient

11. **What is the main difference between triaging in disaster management (DM) and emergency management (EM)?**

Mark only one oval.

- The level of training and experience of the healthcare providers performing the triage
- The availability of medical equipment and resources
- The location of the triage area
- The type of injuries or illnesses being treated
- The urgency and priority of treatment for each patient

12. **What is the purpose of secondary triaging in a medical emergency?** *

Mark only one oval.

- To re-evaluate patients who were not initially prioritized for treatment
- To transfer patients to another medical facility for specialized care
- To gather more information about a patient's medical history and current condition
- To provide additional medical treatment to patients who have already been triaged
- To assess the effectiveness of the triage process and make necessary adjustments

13. **In the triage system, which color is typically associated with the most severe priorit level**

Mark only one oval.

- Green
- Blue
- Yellow
- Red
- Black

14. **In a mass casualty incident (MCI), who is typically responsible for declaring a patier deceased?**

Mark only one oval.

- The first responder on the scene
- The attending physician at the hospital
- The medical examiner
- The patient's family members
- The triage officer

15. **What is the role of the Ministry of Health (MOH) in disaster management?** *

Mark only one oval.

- To provide immediate emergency medical care to victims of a disaster
- To coordinate the response efforts of healthcare providers and facilities in the affected area
- To assess the effectiveness of disaster preparedness plans and make recommendations fo improvement
- To provide financial assistance to victims of a disaster and their families
- To conduct research on disaster medicine and develop new treatment protocols

16. **What is an MCI plan in disaster management?** *

Mark only one oval.

- A plan for providing mental health services to victims of a disaster
- A plan for managing medical emergencies in a hospital or healthcare facility
- A plan for responding to natural disasters such as hurricanes and earthquakes
- A plan for preventing the spread of infectious diseases in a community
- A plan for coordinating the efforts of multiple agencies and organizations in response to a mass casualty incident

17. **What is the role of hospitals in disaster preparedness?** *

Mark only one oval.

- To coordinate the response efforts of healthcare providers and facilities in the affected area
- To provide immediate emergency medical care to victims of a disaster
- To assess the effectiveness of disaster preparedness plans and make recommendations fo improvement
- To establish and maintain emergency management plans and protocols to ensure continuity of patient care during and after a disaster
- To conduct research on disaster medicine and develop new treatment protocols

18. **Which means of communication is considered the most important in disaster management?**

Mark only one oval.

- Telephone communication
- Radio communication
- Social media communication
- Face-to-face communication
- Email communication

19. **During a disaster, who is responsible for overall coordination and control?** *

Mark only one oval.

- The fire department
- The emergency medical services (EMS)
- The local government or emergency management agency
- The military
- The police department

20. **What is the primary role of an incident commander during a disaster?** *

Mark only one oval.

- To coordinate communication between agencies
- To make policy decisions regarding disaster response
- To lead search and rescue efforts
- To oversee overall response and control of the incident
- To provide medical care to victims

21. **Which of the following options correctly defines PTSD?** *

Mark only one oval.

- A physical disorder caused by exposure to chemicals or radiation
- A genetic disorder that affects the body's ability to fight infections
- A disorder caused by the excessive use of drugs or alcohol
- A neurological disorder caused by head injuries or stroke
- A mental disorder caused by the experience of a traumatic event

22. **What are some treatment modalities that may be used to address stress and trauma during and after a disaster?**

Mark only one oval.

- Chemotherapy
- Dialysis
- Surgery
- Acupuncture
- Cognitive-behavioral therapy

23. **Which hospitals were damaged during the Beirut explosion in August 2020?** *

Mark only one oval.

- Lebanese American University Medical Center
- Saint George Hospital University Medical Center
- Rafik Hariri University Hospital
- All of the above
- None of the above

24. Which of the following has been recognized as one of the most significant impacts of the COVID-19 pandemic worldwide in the context of disaster management ?

Mark only one oval.

- Increased mortality rates and strain on healthcare systems
- Disruption of education and social isolation
- Restrictions on travel and tourism
- Increased political polarization
- Economic recession and job loss

This content is neither created nor endorsed by Google.
