# Supplementary material for: Disaster Medicine Training for Medical Students in Lebanon: Quasi-Experimental Comparison of e-Learning and Face-to-Face Modalities
Source: JMIR Med Educ. 2026 Jan 28;12:e80409. doi: 10.2196/80409 (PMC12895154; doi:10.2196/80409)
Supplement: Multimedia Appendix 2 [file mededu_v12i1e80409_app2.docx]

| **Evaluation** | **Choices of the Likert scale** | | | | |
| --- | --- | --- | --- | --- | --- |
|  | Strongly disagree  1 | Disagree  2 | Neutral  3 | Agree  4 | Strongly agree 5 |
| The course met my expectations |  |  |  |  |  |
| The course was organized |  |  |  |  |  |
| The course duration was right |  |  |  |  |  |
| The course urged me to search the elaborated topics |  |  |  |  |  |
| The facilitator is friendly |  |  |  |  |  |
| The course was explained vividly |  |  |  |  |  |
| The facilitator engages us during the explanation |  |  |  |  |  |
| The course enhanced my knowledge of the subject |  |  |  |  |  |
| I would recommend this course to my colleagues |  |  |  |  |  |
| The course is relevant to my carrier |  |  |  |  |  |
| Do you feel more confident about dealing with disasters after this course? |  |  |  |  |  |
| I participate in one of the following educational activities on regular basis continuing education classes, seminars, or conferences dealing with disaster preparedness. |  |  |  |  |  |
| I know who to contact (chain of command) in disaster situations in my community. |  |  |  |  |  |
| I have participated in emergency plan drafting and emergency planning for disaster situations in my community. |  |  |  |  |  |
| I participate in disaster drills or exercises at my workplace (clinic, hospital, etc.) on a regular basis. |  |  |  |  |  |
| I have a list of contacts in the medical or health community in which I practice. I know referral contacts in case of a disaster situation (health department, e.g.,). |  |  |  |  |  |
| Finding relevant information about disaster preparedness related to my community needs is an obstacle to my level of preparedness |  |  |  |  |  |
| I consider myself prepared for the management of disasters. |  |  |  |  |  |
| I know where to find relevant research or information related to disaster preparedness and management to fill in gaps in my knowledge. |  |  |  |  |  |
| I find that the research literature on disaster preparedness and management is easily accessible. |  |  |  |  |  |
| I find that the research literature on disaster preparedness is understandable. |  |  |  |  |  |
| I am familiar with the local emergency response system for disasters. |  |  |  |  |  |
| I would feel confident providing education on coping skills and training for patients who experience traumatic situations so they are able to manage themselves |  |  |  |  |  |
| I am able to discern the signs and symptoms of acute stress disorder and post-traumatic stress syndrome (PTSD) |  |  |  |  |  |
| Evaluation | Open questions | | | | |
| What was the good of this course? |  | | | | |
| What can be improved in the future? |  | | | | |
| Any additional comments? |  | | | | |

### References

35. Glasgow RE, Emmons KM. How can we increase translation of research into practice? Types of evidence needed. Annu Rev Public Health. 2007;28:413-433. doi:10.1146/annurev.publhealth.28.021406.144145. PMID:17150029.

37. French DP, Miles LM, Elbourne D, et al; MERIT Collaborative Group. Reducing bias in trials from reactions to measurement: the MERIT study including developmental work and expert workshop. NIHR Journals Library; 2021. Accessed October 3, 2024. https://www.ncbi.nlm.nih.gov/books/NBK574084/
