# Supplementary material for: Disaster Medicine Training for Medical Students in Lebanon: Quasi-Experimental Comparison of e-Learning and Face-to-Face Modalities
Source: JMIR Med Educ. 2026 Jan 28;12:e80409. doi: 10.2196/80409 (PMC12895154; doi:10.2196/80409)
Supplement: Multimedia Appendix 3 [file mededu_v12i1e80409_app3.docx]

| **Evaluation** | **Choices of the Likert scale** | | | | |
| --- | --- | --- | --- | --- | --- |
|  | Strongly disagree  1 | Disagree  2 | Neutral  3 | Agree  4 | Strongly agree 5 |
| Confident to take care of patients without supervision |  |  |  |  |  |
| Confident to educate patients on stress related to injury |  |  |  |  |  |
| Confident to be triage practitioner and establish temporary health service in disaster situations |  |  |  |  |  |
| Confident as a manager or coordinator in shelter. |  |  |  |  |  |
| Able to recognize the signs and symptoms of acute stress disorder and post-traumatic stress disorder (PTSD) |  |  |  |  |  |
| Confident to perform as a first responder |  |  |  |  |  |
| Confident to perform health assessment in case of bioterrorism |  |  |  |  |  |
| Familiar to perform role of a practitioner in a post-disaster situation |  |  |  |  |  |
| Confident to implement emergency plan and evacuation |  |  |  |  |  |
| Managing emotional outcomes for acute stress disorder or PTSD |  |  |  |  |  |
| Familiar with the roles of organizations in disaster response |  |  |  |  |  |
| Familiar to perform health assessment for PTSD |  |  |  |  |  |
| Participate in peer evaluation of skills on disaster preparedness and response. |  |  |  |  |  |
| Able to describe role in response phase |  |  |  |  |  |
| Familiar with psychological treatment |  |  |  |  |  |
| Confident to perform isolation procedure to reduce risk of community exposure |  |  |  |  |  |
| Know how to operate the decontamination procedures |  |  |  |  |  |
| Know how to use personal protective equipment |  |  |  |  |  |
| Familiar with the triage principles in disaster situations |  |  |  |  |  |
| Have personal/family emergency plans |  |  |  |  |  |
| Familiar with the local emergency response system |  |  |  |  |  |
| Have an agreement with family members |  |  |  |  |  |
| Able to manage the common reactions of disaster survivors |  |  |  |  |  |
| Participated in emergency plans in community |  |  |  |  |  |
| Participated in educational activities about disaster preparedness |  |  |  |  |  |
| Participated in creating an emergency plan for improvements on the local or national level |  |  |  |  |  |
| Read journals related to disaster preparedness |  |  |  |  |  |
| Be considered a key leader in the community in a disaster situation |  |  |  |  |  |
| Know the chain of command in the community |  |  |  |  |  |
| Have a list of contacts in medical centers or community health centers |  |  |  |  |  |
| Know the limitations in knowledge and skills about disaster situations |  |  |  |  |  |
| Know sources of information related to disaster preparedness |  |  |  |  |  |
| Interested in classes about disaster preparedness in the community |  |  |  |  |  |
| The research literature on disaster preparedness is easily accessible |  |  |  |  |  |
| Understand research literature on disaster preparedness |  |  |  |  |  |

### References

35. Glasgow RE, Emmons KM. How can we increase translation of research into practice? Types of evidence needed. Annu Rev Public Health. 2007;28:413-433. doi:10.1146/annurev.publhealth.28.021406.144145. PMID:17150029.

36. Corpuz JCG. A dignified death: management of dead bodies during COVID-19. J Public Health (Oxf). 2022;44(2):e281-e282. doi:10.1093/pubmed/fdab182. PMID:34096608; PMCID:PMC8344556.
