# Supplementary material for: Disaster Medicine Training for Medical Students in Lebanon: Quasi-Experimental Comparison of e-Learning and Face-to-Face Modalities
Source: JMIR Med Educ. 2026 Jan 28;12:e80409. doi: 10.2196/80409 (PMC12895154; doi:10.2196/80409)
Supplement: Multimedia Appendix 4 [file mededu_v12i1e80409_app4.docx]

| ID | Learning Modality | Year | Pre-course Assessment | Post-course Assessment 1 | Post-course Assessment 2 | Confidence Level | Satisfaction Level |
| --- | --- | --- | --- | --- | --- | --- | --- |
|  |  |  | Q1 | Q1 | Q1 | Q1 | Q1 |
|  |  |  | Q2 | Q2 | Q2 | Q2 | Q2 |
|  |  |  | Q3 | Q3 | Q3 | Q3 | Q3 |
|  |  |  | .. | .. | .. | .. | .. |
