# Supplementary material for: Disaster Medicine Training for Medical Students in Lebanon: Quasi-Experimental Comparison of e-Learning and Face-to-Face Modalities
Source: JMIR Med Educ. 2026 Jan 28;12:e80409. doi: 10.2196/80409 (PMC12895154; doi:10.2196/80409)
Supplement: Multimedia Appendix 5 [file mededu_v12i1e80409_app5.docx]

|  | **Pre test** | **Post test** | **Post 1 Month** | **Mean diff**  **(post-pre)** | **P-value within groups** | **Post 1 Month Pre** | **P-value within groups** |
| --- | --- | --- | --- | --- | --- | --- | --- |
| **Overall Sample** |  |  |  |  |  |  |  |
| **F2F Exp Mean (SD)** | 6.3(2.3) | 12.3(1.8) | 14.3(2.4) | 5.9(3.0) | **<0.001** | 7.9(3.1) | **<0.001** |
| **F2F Cont Mean (SD)** |  | 12.2(1.7) | 15.7(3.9) |  |  |  |  |
| **E-learning Exp Mean (SD)** | 6.6(2.3) | 12.0(2.1) | 14.6(3.5) | 5.4(3.4) | **<0.001** | 8(4.3) | **<0.001** |
| **E-learning Cont Mean (SD)** |  | 13.5(3.7) | 16.4(4.4) |  |  |  |  |
| **P-value between groups** | 0.602 | 0.404 | 0.644 |  |  |  |  |
| **Second year** |  |  |  |  |  |  |  |
| **F2F Exp Mean (SD)** | 6.1(2.9) | 11.7(2.4) | 13.7(3.8) | 5.6(4.2) | **0.039** | 7.6(3.1) | **0.002** |
| **F2F Cont Mean (SD)** |  | 12.4(0.9) | 16.4(3.2) |  |  |  |  |
| **E-learning Exp Mean**  **(SD)** | 6.7(3.4) | 10.7(2.6) | 13.7(4.2) | 4(5.6) | 0.741 | 7(4.3) | 0.144 |
| **E-learning Cont Mean (SD)** |  | 11.7(2) | 15.7(3.9) |  |  |  |  |
| **P-value** | 0.761 | 0.553 | 0.989 |  |  |  |  |
| **Third Year** |  |  |  |  |  |  |  |
| **F2F Exp Mean (SD)** | 6.8(1.4) | 12.1(2.0) | 14.1(2.1) | 5.3(2.1) | **<0.001** | 7.3(2.3) | **<0.001** |
| **F2F Cont Mean (SD)** |  | 12.7(1.3) | 16.8(4.6) |  |  |  |  |
| **E-learning Exp Mean (SD)** | 6.3(2.0) | 12.1(2.5) | 14.6(3.8) | 5.8(3.2) | **<0.001** | 8.3(4.3) | **<0.001** |
| **E-learning Cont Mean (SD)** |  | 11.6(2.9) | 16.5(4.5) |  |  |  |  |
| **P-value** | 0.387 | 0.920 | 0.713 |  |  |  |  |
| **Fourth Year** |  |  |  |  |  |  |  |
| **F2F Exp Mean (SD)** | 6.1(2.9) | 13.3(1.1) | 15.1(2.6) | 7.1 (3.6) | **0.006** | 9.0 (3.9) | **0.003** |
| **F2F Cont Mean (SD)** |  | 12.2(0.9) | 16.4(3.2) |  |  |  |  |
| **E-learning Exp Mean (SD)** | 7.0(2.5) | 12.2(1.9) | 14.3(3.8) | 4.9(3.3) | **<0.001** | 7.2(4.8) | **<0.001** |
| **E-learning Cont Mean**  **(SD)** |  | 16.1(4.5) | 16.6(4.5) |  |  |  |  |
| **P-value** | 0.378 | 0.117 | 0.613 |  |  |  |  |
| **Fifth Year** |  |  |  |  |  |  |  |
| **F2F Exp Mean (SD)** | 6.1(2.6) | 12.2(1.7) | 14.3(1.9) | 6.1(2.7) | **0.005** | 8.2(3.4) | **<0.001** |
| **F2F Cont Mean (SD)** |  | 11.7(2.2) | 15.5(4.1) |  |  |  |  |
| **E-learning Exp Mean (SD)** | 5.7(1.5) | 11.8(2.2) | 15.9(1.7) | 6.1(3) | **0.005** | 10.1(2.6) | **<0.001** |
| **E-learning Cont Mean (SD)** |  | 13.4(1.8) | 16.2(4.6) |  |  |  |  |
| **P-value** | 0.737 | 0.680 | 0.086 |  |  |  |  |

*SD Standard deviation, P-value less than 0.05 is considered significant*
